# Efficacy and Safety of Cabozantinib in Patients with Advanced or Metastatic Renal Cell Carcinoma: A Multicenter Retrospective Cohort Study

**DOI:** 10.3390/biomedicines10123172

**Published:** 2022-12-07

**Authors:** Koji Iinuma, Risa Tomioka-Inagawa, Koji Kameyama, Tomoki Taniguchi, Kei Kawada, Takashi Ishida, Shingo Nagai, Torai Enomoto, Shota Ueda, Makoto Kawase, Shinichi Takeuchi, Kota Kawase, Daiki Kato, Manabu Takai, Keita Nakane, Takuya Koie

**Affiliations:** 1 Department of Urology, Gifu University Graduate School of Medicine, Gifu 501-1194, Japan; 2 Department of Urology, Kizawa Memorial Hospital, 590 shimokobi, Kobicho, Minokamo, Gifu 505-8503, Japan; 3 Department of Urology, Ogaki Municipal Hospital, 4-86 Minaminokawa-cho, Ogaki, Gifu 503-8502, Japan; 4 Department of Urology, Gifu Prefectural General Medical Center, 4-6-1 Noisiki, Gifu 500-8717, Japan; 5 Department of Urology, Gifu Municipal Hospital, 7-1 Kashimacho, Gifu 500-8513, Japan; 6 Department of Urology, Toyota Memorial Hospital, 1-1 Heiwacho, Toyota, Aichi 471-8513, Japan; 7 Department of Urology, Matsunami General Hospital, 185-1 Kasamatsucho, Hashima-gun, Gifu 501-6062, Japan; 8 Department of Urology, Japanese Red Cross Takayama Hospital, 3-113-11 Tenman-machi, Takayama-shi, Gifu 506-8550, Japan

**Keywords:** cabozantinib, renal cell carcinoma, Japanese patients, C-reactive protein, prior tyrosine kinase inhibitors

## Abstract

A multicenter retrospective study was conducted to evaluate the efficacy and safety of cabozantinib in patients with advanced or metastatic renal cell carcinoma (mRCC). We enrolled 53 patients with mRCC who received cabozantinib at eight institutions in Japan. The primary endpoint was overall survival (OS). The secondary endpoints were objective response rate (ORR), disease control rate (DCR), and progression-free survival (PFS). In addition, we analyzed prognostic factors in patients with mRCC treated with cabozantinib. The median follow-up period was 8 months, and the median OS was 20.0 months. The ORR and DCR were 39.6% and 83.0%, respectively. The median PFS was 11.0 months. PFS was significantly shorter in patients previously treated with at least two tyrosine kinase inhibitors and in those with C-reactive protein (CRP) ≥ 1.27 mg/dL (*p* = 0.021 and *p* = 0.029, respectively). Adverse events of any grade and grades ≥3 occurred in 42 (79.2%) and 10 (18.9%) patients, respectively. Cabozantinib is a useful treatment option for patients with mRCC and may benefit from earlier use. In this study, CRP ≥ 1.27 mg/dL is a poor prognostic factor in patients treated with cabozantinib, and careful follow-up may be required in treating patients with high CRP.

## 1. Introduction

In a 2019 report, renal cell carcinoma (RCC) was the 10th most commonly diagnosed cancer in men and the 17th most frequently diagnosed cancer in women in Japan [1]. While most detected RCCs are still localized and treated surgically, one-third of cases are diagnosed as advanced or metastatic RCC (mRCC) [2]. In addition, 20–50% of patients with RCC who undergo surgery develop metastatic disease [2]. The initial management of mRCC varies according to prognostic factors [2].

Tyrosine kinase inhibitors (TKIs) are the standard therapy for mRCC. However, this has changed dramatically with the advent of immune checkpoint inhibitors (ICIs), such as nivolumab (NIVO; a programmed cell death protein 1 (PD-1) inhibitor), pembrolizumab (PD-1 inhibitor), avelumab (PD-1 ligand 1 inhibitor), and ipilimumab (IPI; an anti-cytotoxic T lymphocyte antigen 4 [CTLA-4] monoclonal antibody) [3]. According to the results of randomized phase III trials, including Checkmate 214 [4], KEYNOTE-426 [5], JAVELIN Renal 101 [6], IMmotion151 [7], CheckMate 9ER [8], and CLEAR [9], the combination of ICIs and TKIs offer significant clinical advantages compared with TKI monotherapy. Although combined immunotherapy has become the standard treatment for mRCC in the current era, treatment with ICIs might not confer equivalent clinical benefits in all patients with mRCC. In our previous study, which evaluated the efficacy and safety of NIVO + IPI in patients with mRCC, 18.5–23.5% of patients developed progressive disease (PD) [10,11]. The combination of ICIs and TKIs resulted in disease progression in approximately half of the cases at 2 years in phase III trials [5,6,8,9]. Therefore, subsequent therapy after ICI therapy is important. We previously reported that TKIs might be a useful secondary treatment option after the discontinuation of NIVO + IPI [12]. TKIs remain an important treatment for mRCC in the ICI era.

The common histological type of RCC is clear cell RCC [13,14], which is commonly showing mutations in the tumor suppressor Von Hippel-Lindau gene, triggering a decrease in the degradation of hypoxia-inducible factor and increasing the vascular endothelial growth factor (VEGF) transcription and tumor angiogenesis [15]. TKIs that target the VEGF receptor (VEGFR) have been used. However, resistance can occur because of the upregulation of alternative signaling pathways that promote angiogenesis and invasion, such as the MET and AXL pathways [16].

Cabozantinib is a multiple-receptor TKI targeting MET (c-MET), VEGFR2, RET, AXL, KIT, and TIE-2, all implicated in tumor growth, metastasis, and angiogenesis [17]. Choueiri et al. reported the results of a phase III, randomized, open-label METEOR study (NCT01865747) that compared the efficacy and safety of cabozantinib versus everolimus in patients with mRCC who showed disease progression after previous VEGFR–TKI therapy. The progression-free survival (PFS) and overall survival (OS) were significantly better in patients treated with cabozantinib than in patients treated with everolimus (7.4 vs. 3.8 months; hazard ratio (HR) 0.51, 95% CI 0.41–0.62, *p* < 0.0001, 21.4 vs. 16.5 months, *p* = 0.00026, respectively), along with favorable objective response rate (ORR) (17% vs. 3%; *p* < 0.0001) [18,19]. In the phase II CABOSUN trial (NCT01835158), cabozantinib also showed a PFS benefit (8.2 vs. 5.6 months) and reduced the rate of disease progression or death by 34% (HR 0.66, 95% confidence interval [CI] 0.46–0.95, *p* = 0.012) over first-line sunitinib [20]. Tomita et al. demonstrated the efficacy and safety of cabozantinib through a bridging study to METEOR in Japanese patients with mRCC who showed disease progression after prior TKI therapy [21]. Of the 35 patients enrolled, the ORR was 20.0% (95%CI 9.8–34.3%), and the clinical benefit rate was 85.7% (95% CI, 69.7–95.2%) [21]. However, there are few reports of Japanese patients, including those who used cabozantinib as the first-line treatment. Therefore, a multicenter retrospective study was conducted to evaluate the efficacy and safety of cabozantinib in patients with histologic types of clear cell and nonclear cell mRCC. In addition, we evaluated the predictive factors for progressive disease during cabozantinib administration.

## 2. Materials and Methods

### 2.1. Patients

We conducted a retrospective multicenter cohort study of patients with mRCC treated with cabozantinib at eight institutions in Japan between May 2020 and September 2022. Patients were stratified into favorable-, intermediate-, or poor-risk groups according to the International Metastatic Renal Cell Carcinoma Database Consortium (IMDC) risk model [22]. The collected clinicopathological data included age, sex, Eastern Cooperative Oncology Group (ECOG) performance status (PS) [23], primary IMDC risk classification, histology, C-reactive protein (CRP) level, surgical history, number of treatment regimens before administration of cabozantinib, number of TKI agents before administration of cabozantinib, prior therapy with ICI agents before administration of cabozantinib, number and location of metastatic sites.

This study was approved by the Institutional Review Board of Gifu University (approval number: 2020-271) and respective institutional review boards. Patient consent was not required because of the retrospective nature of the study. The provisions of the ethics committee and the ethics guidelines in Japan did not require written consent because the study information was disclosed to the public in case of retrospective and/or observational studies using materials such as existing documentation. Details of the study can be accessed at https://www.med.gifu-u.ac.jp/visitors/disclosure/docs/2020-271.pdf (accessed on 3 March 2021).

### 2.2. Treatment Schedule of Cabozantinib

The cabozantinib treatment dose was determined at the treatment institution. Treatment was continued until disease progression according to radiological evaluation or unacceptable toxicity for treatment-related adverse events (TRAEs).

### 2.3. Patient Evaluation

Baseline evaluations before the administration of cabozantinib included a complete history and physical examination, including chest, abdominal, and pelvic computed tomography (CT) and/or magnetic resonance imaging (MRI). In addition, the American Joint Committee on Cancer Staging Manual Tumor staging was used to determine the tumor stage [24].

All patients underwent CT or MRI every 1–3 months until disease progression according to radiological evaluation or treatment discontinuation for TRAEs. Based on the Response Evaluation Criteria in Solid Tumors (RECIST) guidelines (version 1.1) [25], the best overall response (BOR) was defined as complete response (CR), partial response (PR), stable disease (SD), or PD. The ORR was the proportion of patients who achieved CR or PR, and the disease control rate (DCR) was the proportion of patients with CR, PR, or SD.

### 2.4. Safety

TRAEs were graded according to the National Cancer Institute Common Terminology Criteria for Adverse Events, version 5.0 [26] and reported at initiation and at least 100 days after the last administration of cabozantinib.

### 2.5. Statistical Analysis

The primary endpoint was OS. The secondary endpoints were ORR, DCR, BOR, and PFS. Follow-up duration was defined as the interval from the date of cabozantinib initiation to the last follow-up examination or the documented date of death, whichever occurred first. OS was defined as the interval between cabozantinib initiation and death. PFS was defined as the interval from treatment initiation to the first RECIST-defined disease progression or death, whichever occurred earlier. OS and PFS were estimated using the Kaplan–Meier method. Differences were assessed according to clinical variables using a log-rank test. The Cox proportional hazards regression model assessed the clinical parameters for predicting progression-free survival. The cutoff value of CRP was determined using receiver operating characteristic curve analysis [27]. Patients were classified into two groups based on median age. Data were analyzed using the JMP 14 (SAS Institute Inc., Cary, NC, USA) software.

## 3. Results

### 3.1. Patients

Between May 2020 and September 2022, 53 patients with mRCC were treated with cabozantinib at 8 institutions in Japan. The demographic data of the enrolled patients are presented in Table 1. ECOG PS ≥ 2 was observed in 18 patients (33.9%). The median follow-up period from initiation of cabozantinib to the date of analysis or death was 8 months (interquartile range (IQR): 3.5–14.0).

### 3.2. Treatment Dose of Cabozantinib

The starting dose of cabozantinib was 60 mg in 8 patients (15.1%), 40 mg in 28 patients (52.8%), and 20 mg in 17 patients (32.1%).

### 3.3. Efficacy and Oncological Outcomes

**Figure 1:**
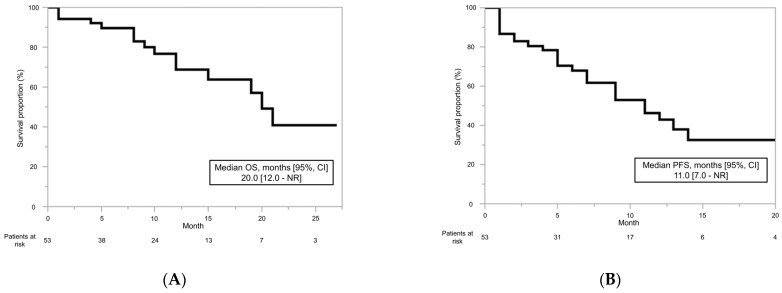
Overall survival (OS) and progression-free survival (PFS) for cabozantinib in patients with advanced or metastatic renal cell carcinoma. The median OS was 20.0 months (**A**). The median PFS was 11.0 months (**B**).

The median OS for the primary endpoint was 20 months (95% CI: 12.0–not reached (NR)) (Figure 1A). The median PFS was 11.0 months (95% CI: 7.0–NR) (Figure 1B). The treatment effects in the patients who received cabozantinib are shown in Table 2. Regarding the BOR, no patients achieved CR, 21 patients (39.6%) achieved PR, and 23 patients (43.4%) showed SD. The ORR and DCR were 39.6% and 83.0%, respectively.

**Figure 2:**
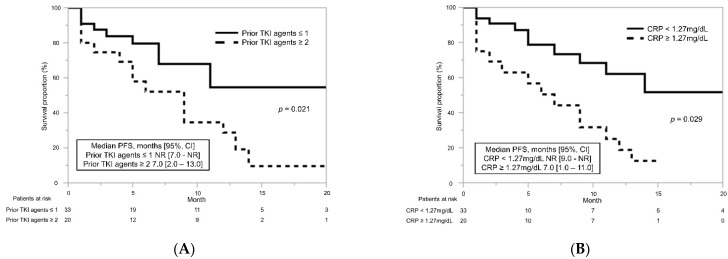
Progression-free survival (PFS) for cabozantinib in patients with advanced or metastatic renal cell carcinoma. (**A**) PFS for cabozantinib stratified by the number of prior tyrosine kinase inhibitor agents. (**B**) PFS for cabozantinib stratified by C-reactive protein (CRP) level.

According to the number of previous TKI treatments, the median PFS in patients with at least two TKIs was significantly shorter than that in patients with less than one TKI (7.0 months vs. NR, *p* = 0.021) (Figure 2A). Based on CRP stratification, the median PFS was significantly shorter in patients with CRP ≥ 1.27 mg/dL than with CRP < 1.27 mg/dL (7.0 months vs. NR, *p* = 0.029) (Figure 2B). There was no significantly association with PFS by patient age (*p* = 0.88), gender (*p* = 0.09), or IMDC risk classification (*p* = 0.18). In univariate and multivariate analyses, previous treatments with at least two TKIs and CRP ≥ 1.27 mg/dL were associated with poor PFS (Table 3).

### 3.4. Safety

TRAEs are presented in Table 4. Four patients (7.6%) discontinued treatment owing to TRAEs: proteinuria in 2 patients, rectal ulcer in 1, and erythema multiforme in 1 patient. None of the patients died of TRAEs during the follow-up period.

## 4. Discussion

Patients at all risks in the IMDC risk classification and those with no prior TKI treatment were included in this study. The median OS and PFS of this study were 20.0 months and 11.0 months, respectively. The ORR and DCR were 39.6% and 83.0%, respectively. The METEOR trial was a randomized phase III trial that compared the efficacy and safety of cabozantinib versus everolimus in patients with mRCC who showed disease progression after previous TKI treatment [18,19]. Choueiri et al. reported that PFS and OS were significantly better in patients treated with cabozantinib than in patients treated with everolimus (7.4 vs. 3.8 months; HR 0.51, 95% CI: 0.41–0.62, *p* < 0.0001, 21.4 vs. 16.5 months, *p* = 0.00026, respectively) [18,19]. The ORR was also favorable in patients treated with cabozantinib compared with that in patients treated with everolimus (17% vs. 3% [2,3,4,5,6], *p* < 0.0001) [18,19]. In the CABOSUN trial evaluating cabozantinib compared with sunitinib as first-line therapy in patients with mRCC, eligible patients had untreated clear cell mRCC, ECOG PS of 0–2, and intermediate- or poor-risk per IMDC risk classification [20]. In this study, cabozantinib demonstrated a significant clinical benefit in PFS (8.2 vs. 5.6 months, *p* = 0.012) and ORR (33% vs. 12%) over sunitinib as first-line therapy in patients with intermediate- or poor-risk mRCC [20]. Tomita et al. reported the results of a bridging study to METEOR in Japanese patients with mRCC who showed disease progression after prior TKI therapy [21]. Of the 35 patients enrolled, ORR and DCR were 20.0% (90% CI: 9.8–34.3%) and 85.7% (95% CI: 69.7–95.2%), respectively, and the 6-month PFS was 72.3% [21]. As the real-world data, Albiges et al. reported treatment patterns and outcomes for patients treated with cabozantinib through the French Early Access Program [28]. Among 410 patients, the median OS was 14.4 months, and the 12-month OS rate was 56.5% (95% CI: 51.5–61.2) [28]. Our results are comparable to those in the above studies, suggesting that cabozantinib may be effective in Japanese patients with mRCC in clinical practice.

Regarding safety, in this study, 10 patients (18.9%) experienced grades 3–4 TRAEs, and four patients (7.6%) discontinued cabozantinib because of TRAEs. Compared to the METEOR [18,19] and CABOSUN trials [20], our results showed fewer results for grade 3 or higher TRAEs. Tomonari et al. analyzed the clinical outcomes of cabozantinib in unresectable hepatocellular carcinoma and compared treatment outcomes between full- (60 mg) and reduced-dose (20–40 mg) groups [29]. The ORR and DCR were not significantly different between the two groups [29]. The incidence of TRAEs, such as decreased appetite, fatigue, and diarrhea, was significantly higher in the full-dose group than in the reduced-dose group for all grades (*p* < 0.05) [29]. In our study, only 15.1% of patients started cabozantinib at 60 mg, but the efficacy was comparable to that in previous studies, with fewer grade 3 or higher TRAEs, suggesting that starting cabozantinib at a reduced dose may be a safe treatment option for Japanese patients with mRCC.

There are limited data on the efficacy of cabozantinib as a treatment line. Gan et al. revealed the results of a multicenter retrospective study in patients with mRCC treated with cabozantinib across the first to fourth line [30]. They reported that the ORR and time to treatment failure of cabozantinib were maintained from the first- to fourth-line settings [30]. In our study, the median PFS in patients treated with at least two TKIs was significantly shorter than that in patients who were treated with one TKI (7.0 months vs. NR, *p* = 0.021). Additionally, previous treatments with at least two TKIs were associated with poor PFS in univariate and multivariate analyses. Although cabozantinib treatment is controversial, our results suggest that cabozantinib may be more effective when used early.

CRP is a clinicopathological marker of systemic inflammation and immune activation and can be readily measured in peripheral blood samples. Elevated CRP level is a poor prognostic marker in many cancers, including mRCC [31,32,33,34,35,36]. Beuselinck et al. reported that the baseline CRP level was a strong independent variable linked with the ORR, PFS, and OS in patients with mRCC treated with sunitinib [35]. Our results revealed that a CRP ≥ 1.27 mg/dL was a risk factor for disease progression. Based on our results, CRP could be used as a prognostic biomarker in patients with mRCC. The cutoff value for CRP ranged from 1.5 to 11 mg/dL depending on the report, and a consensus cutoff value may be needed in the future [31,32,33,34,35,36].

CRP at the start of treatment is also an important prognostic factor, although changes in CRP after treatment have been reported to affect prognosis [37,38,39]. Saito et al. showed that mRCC patients whose CRP was normal at the start of treatment or normalized during the course of treatment had a significantly better prognosis than patients whose CRP was elevated at the start of treatment and did not normalize during the course of treatment [37]. Teishima et al. reported that CRP levels after cytoreductive nephrectomy might affect the OS in patients with mRCC who received TKIs [38]. In ICI era, Ishihara et al. reported that changes of CRP in the early phase of nivolumab treatment were significantly associated with survival in patients with mRCC [39]. Although CRP at the start of treatment was used for analysis in this study, posterior changes in CRP could be evaluated to more accurately predict the prognosis of mRCC patients receiving cabozantinib.

In addition to CRP, copeptin and apelin have been reported as biomarkers of kidney disease [40,41]. Copeptin is measured as an alternative to vasopressin, which plays a detrimental role in autosomal dominant polycystic kidney disease (ADPKD), and apelin regulates angiogenesis and stimulates endothelial cell growth and migration [40,41]. It is known that ADPKD patients have higher copeptin levels and lower apelin levels compared to healthy individuals [40]. Regarding the association with cancer, patients with malignant neoplasms had higher apelin levels compared to healthy subjects and were also closely related to the stage of the disease [41]. Biomarkers may be needed that are effective in predicting the efficacy of drug therapy and serve as indicators of treatment response for mRCC.

Our study has several limitations. First, this was a retrospective study conducted using multicenter data. Therefore, this study has an inherent potential for bias, with diagnostic and therapeutic variations among the institutions. Second, a relatively small number of patients were enrolled, and the follow-up period was relatively short. Third, because we enrolled patients with mRCC who received cabozantinib on various lines, no control group patients received other TKIs for mRCC. Fourth, the study included papillary renal cell carcinoma, Xp11.2 translocation renal cell carcinoma, and fumarate renal cell carcinoma as well as clear cell renal cell carcinoma, and 24.5% had an unknown histologic type. Despite the above limitations, the oncological outcomes and safety signals were equivalent to those of other studies. Therefore, cabozantinib may have potential advantages for patients with mRCC, leading to positive treatment effects. Previous treatment with at least two TKIs and CRP ≥ 1.27 mg/dL were risk factors for disease progression during cabozantinib treatment. Further studies and long-term evaluations are required to determine the efficacy of cabozantinib.

## 5. Conclusions

Here, we retrospectively investigated the efficacy and safety of cabozantinib in patients with mRCC. The oncological outcomes and safety signals of cabozantinib were equivalent to those in previous studies. Therefore, cabozantinib is a useful treatment option for patients with mRCC and may benefit from earlier use. In our study, CRP ≥ 1.27 mg/dL is a poor prognostic factor, and elevated CRP may be a poor prognostic factor in patients treated with cabozantinib; such patients may require a more careful follow-up.

## Figures and Tables

**Table 1:** Patient characteristics at the start of cabozantinib treatment.

Covariates	
Age (year, median, interquartile range)	72.0 (67.0–78.5)
Gender (number, %)	
Male	44 (83.0)
Female	9 (17.0)
The Eastern Cooperative Oncology Group performance status (number, %)	
0	18 (34.0)
1	17 (32.1)
2	9 (17.0)
3	8 (15.0)
4	1 (1.9)
Primary IMDC risk classification (number, %)	
Favorable	12 (22.6)
Intermediate-risk	17 (51.0)
Poor-risk	14 (26.4)
Histology	
Clear cell renal cell carcinoma	31 (58.5)
Papillary renal cell carcinoma	7 (13.2)
Xp11.2 translocation carcinomas	1 (1.9)
Fumarate hydratase-deficient renal cell carcinoma	1 (1.9)
Unknown	13 (24.5)
C-reactive protein (mg/dL, median, interquartile range)	0.55 (0.14–3.63)
The patients who underwent surgery before administration of cabozantinib (number, %)	37 (69.8)
Number of treatment regimens before administration of cabozantinib (number, %)	
0	8 (15.1)
1	16 (30.2)
2	9 (17.0)
3	8 (15.1)
≥4	12 (22.6)
Number of prior tyrosine kinase inhibitor agents before administration of cabozantinib (number, %)	
0	22 (41.5)
1	11 (20.8)
≥2	20 (37.7)
Prior therapy with anti-PD1, anti-CTLA4, and anti-PD-L1 agents before administration of cabozantinib (number, %)	
Nivolumab	32 (60.4)
Ipilimumab	16 (30.2)
Pembrolizumab	2 (3.8)
Avelumab	1 (1.9)
Number of metastatic sites	
1	17 (32.1)
2	19 (35.8)
3	7 (13.2)
≥4	10 (18.9)
Total number of metastatic sites (number, %)	
Lung	38 (71.7)
Lymph node	23 (43.4)
Bone	16 (30.2)
Liver	8 (15.1)
Adrenal gland	7 (13.2)
Pancreas	6 (11.3)
Others	14 (26.4)

CTLA-4, cytotoxic T-lymphocyte-associated protein 4; IMDC, International Metastatic Renal Cell Carcinoma Database Consortium; PD-1, programmed cell death 1; PDL-1, programmed cell death ligand 1.

**Table 2:** Treatment effect in patients who received cabozantinib.

	Total (*n* = 53)
Objective response rate (CR + PR, number, %)	21 (39.6)
Disease control rate (CR + PR + SD, number, %)	44 (83.0)
Best overall response (number, %)	
CR	0
PR	21 (39.6)
SD	23 (43.4)
PD	9 (17.0)

CR, complete response; PD, progressive disease; PR, partial response; SD, stable disease.

**Table 3:** Univariate and multivariate analyses of clinical parameters for the prediction of progression-free survival.

		Univariate Analysis			Multivariate Analysis		
	*n*	HR	95% CI	*p*	HR	95% CI	*p*
Age							
<72 years	24	1 (ref.)	-	-	1 (ref.)	-	-
≥72 years	29	0.81	0.27–2.40	0.71	1.24	0.29–5.40	0.77
Gender							
Male	44	1 (ref.)	-	-	1 (ref.)	-	-
Female	9	0.26	0.05–1.40	0.12	0.42	0.04–4.28	0.47
International Metastatic Renal Cell Carcinoma Database Consortium							
Favorable risk	12	1 (ref.)	-	-	1 (ref.)	-	-
Intermediate risk	27	3.40	0.15–4.24	0.09	7.98	1.0–63.9	0.051
Poor risk	14	0.79	0.15–4.24	0.79	2.96	0.32–27.1	0.34
Prior tyrosine kinase inhibitor agents							
≤1	33	1 (ref.)	-	-	1 (ref.)	-	-
≥2	20	6.90	1.97–24.2	0.003	10.4	1.96–54.8	0.006
C-reactive protein							
<1.27 mg/dL	33	1 (ref.)	-	-	1 (ref.)	-	-
≥1.27 mg/dL	20	6.90	1.97–24.2	0.003	11.4	2.26–57.5	0.003

CI, confidence interval; HR, hazard ratio; *n*, number; ref., reference.

**Table 4:** Adverse events for cabozantinib.

Event	All Grades (*n*, %)	Grades 3–4 (*n*, %)
Any events	42 (79.2)	10 (18.9)
Diarrhea	14 (26.4)	2 (3.8)
Hepatobiliary disorders	12 (22.6)	1 (1.9)
Hypertension	11 (20.8)	1 (1.9)
Palmar–plantar erythrodysesthesia syndrome	9 (17.0)	3
Hypothyroidism	9 (17.0)	0
Anemia	3 (5.7)	1 (1.9)
Creatinine increased	3 (5.7)	1 (1.9)
Proteinuria	3 (5.7)	1 (1.9)
Dysgeusia	2 (3.8)	0
Hoarseness	2 (3.8)	0
Erythema multiforme	2 (3.8)	0
Rectal ulcer	1 (1.9)	1 (1.9)
Peripheral sensory neuropathy	1 (1.9)	0
Anorexia	1 (1.9)	0
Periodontal disease	1 (1.9)	0

*n*, number.

## Data Availability

Data and materials are provided in this paper.
